# Adrenal Collision Tumor: Coexistence of Pigmented Adrenal Cortical Oncocytoma and Ganglioneuroma

**DOI:** 10.1155/2016/5790645

**Published:** 2016-12-08

**Authors:** Hye Seung Lee, Yoo Jin Choi, Chungyeul Kim, Baek-Hui Kim

**Affiliations:** Department of Pathology, Korea University School of Medicine, Seoul, Republic of Korea

## Abstract

*Background*. Adrenal collision tumors (ACTs), in which distinct tumors coexist without intermingling in the same adrenal gland, are rare and their actual prevalence is unknown. ACTs commonly consist of adrenal cortical adenoma, pheochromocytoma, or metastatic malignant tumor.* Case Report*. A 32-year-old woman who had been experiencing gastric discomfort for one month was referred to our hospital with abnormal imaging findings. The physical examination and the laboratory data including endocrine studies were unremarkable. Abdomen computed tomography (CT) and magnetic resonance imaging (MRI) showed two adjacent masses in the left suprarenal fossa, and a laparoscopic left adrenalectomy was done. Histological and immunohistochemical (IHC) examinations revealed two distinct tumors: a pigmented adrenal cortical oncocytoma (ACO) and a ganglioneuroma, respectively.* Conclusion*. Both tumors are rare in the adrenal gland and exist as ACTs only exceptionally rarely. This is the first reported case of coexisting oncocytoma and ganglioneuroma in the same adrenal gland to our knowledge.

## 1. Introduction

Most primary adrenal neoplasms are from either cortical cells or medullary chromaffin cells [1]. Adrenal cortical adenomas, which may produce glucocorticoid hormones, are the most common tumor of the adrenal cortex. They are usually nonfunctional but sometimes can be detected by hormonal symptoms, such as Cushing's syndrome, hyperaldosteronism, or virilization [2]. Among nonfunctioning cortical tumors, oncocytomas are considered a variant of adrenal cortical adenomas. Adrenal cortical oncocytomas (ACOs) do not express steroidogenic enzymes, and they share histological features with their analogs at other sites [3]. Usually, they have a good prognosis but, occasionally, they do not [4]. Ganglioneuromas are rare benign tumors composed of mature Schwann cells, nerve fibers, and a variable number of ganglion cells, mainly originating from primordial neural crests [5]. They occur in mediastinal and aortocaval sympathetic ganglia and less frequently in the adrenal gland. Both oncocytomas and ganglioneuromas are mostly found as a single mass [4, 6]. In this paper, we present a case of very rare and first reported collision tumors in the adrenal gland: the coexistence of a pigmented ACO and a ganglioneuroma. This study was approved by the Korea University Guro Hospital Institutional Review Board (KUGH15365-001).

## 2. Case Report

**Figure 1:**
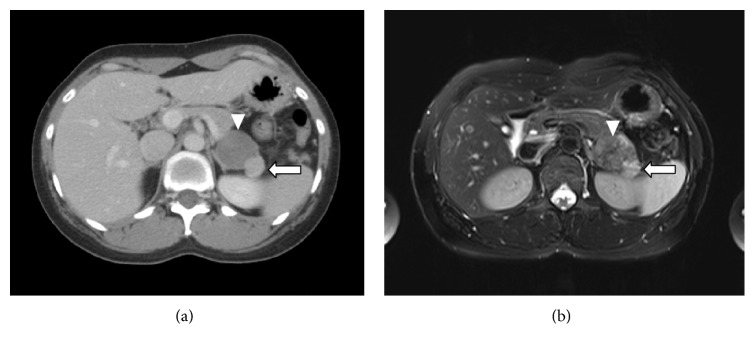
Imaging findings of the patient. (a) Computed tomography shows two adjacent round solid masses in the left suprarenal fossa: a 4 cm low-density mass (arrowhead) and another 1.8 cm enhancing mass (arrow). (b) On T2 magnetic resonance imaging, the 4 cm mass (arrowhead) showed intermediate signal intensity with subtle peripheral and delayed centripetal enhancement in the arterial phase, suggesting a neurogenic tumor. In contrast, the 1.8 cm mass (arrow) showed similar intensity to the spleen.

A 32-year-old Korean woman who had been experiencing gastric discomfort for one month was referred to Korea University Guro Hospital with abnormal imaging findings. Abdominal computed tomography (CT) revealed a 4 cm low-density mass and another 1.8 cm enhancing mass in the left suprarenal fossa, both of which were considered to have originated from the adrenal gland or retroperitoneal soft tissue (Figure 1(a)). She had no medical history or hormonal symptoms, and her vital signs were stable. In addition, her review of systems and physical examination were unremarkable.

Laboratory data showed normal values for complete blood cell counts and chemistry panel results. Endocrine tests were also done, and the results are summarized in Table 1. The catecholamine tests, including norepinephrine, epinephrine, and vanillylmandelic acid (VMA) (plasma and 24 hr urine), were all in the normal range. The serum cortisol level was suppressed to the reference level with low-dose dexamethasone suppression tests. The plasma adrenocorticotropic hormone (ACTH) level was normal. Interestingly, the serum aldosterone and the plasma renin levels increased to 46.5 ng/dL and 15.15 ng/mL/hr, respectively. However, secondary hyperaldosteronism was considered due to the low aldosterone to renin ratio (3.069).

An additional magnetic resonance imaging (MRI) scan showed two abutting masses on the left adrenal gland (Figure 1(b)). T2 imaging revealed a 4 cm intermediate signal intensity lesion. It showed subtle peripheral and delayed centripetal enhancement in the arterial phase, indicating a neurogenic tumor. Another 1.8 cm mass with similar intensity to the spleen was also visualized. Thus, the possibility of an accessory spleen or silent pheochromocytoma was considered. Although neither had any features of malignancy, a laparoscopic left adrenalectomy was performed due to the size of the 4 cm mass.

**Figure 2:**
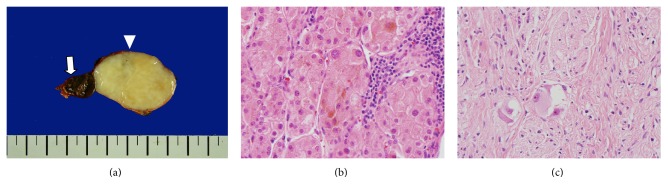
Histological findings of the patient. (a) The gross specimen shows a 2 cm dark-brownish mass (arrow) and a 4.5 cm whitish solid mass (arrowhead) within the adrenal cortex. (b) The 2 cm mass showed abundant eosinophilic, granular cytoplasm with variable sized brown pigments (40x). (c) The 4.5 cm mass showed fascicles of Schwann-like cells and some clusters of ganglion cells (40x).

Grossly, two adjacent but uncombined well-demarcated oval-shaped masses were confined within the yellowish adrenal cortex (Figure 2(a)). The smaller mass (2.0 × 1.8 × 1.2 cm) was mahogany brown in color with a soft bulging appearance. Microscopically, it was composed of compactly arranged polygonal cells with mild-to-moderate nuclear pleomorphism and abundant eosinophilic granular cytoplasm (Figure 2(b)). A considerable amount of brown cytoplasmic pigments was seen in the hematoxylin and eosin stain, which seemed to be lipofuscin rather than melanin. No features of malignancy, such as brisk mitotic activity of more than 5 in 10 in a high-power field of view, coagulative tumor necrosis, capsular invasion, or lymphovascular invasion, were found [4]. On immunohistochemical (IHC) examination, the tumor cells were positive for inhibin-alpha, synaptophysin, and Melan-A but negative for EMA and HMB45. Thus, the tumor was diagnosed as a pigmented ACO.

The larger mass (4.5 × 3.5 × 3.0 cm) was whitish to light yellowish in color, and the consistency was firm (Figure 2(a)). Microscopically, the tumor cells were arranged in loosely fascicular pattern (Figure 2(c)), and they showed wavy nuclei, inconspicuous nucleoli, and elongated cytoplasm. Additionally, a variable number of large cells were admixed with wavy spindle cells. These cells seemed to be ganglion cells, because they had basophilic vesicular nuclei, prominent nucleoli, and abundant cytoplasm. Mitotic activity and coagulative tumor cell necrosis were absent. On IHC examination, both spindle cells and ganglion-like cells were positive for S-100 protein and neurofilament. Accordingly, the cells were considered Schwann cells and mature ganglion cells, respectively, and the mass was diagnosed as a ganglioneuroma.

The postoperative course was uneventful. After one year, physical examination and follow-up CT also showed no remarkable findings.

## 3. Discussion

A collision tumor is said to occur when two or more histologically distinct tumors coexist without admixture at the interface [7]. Since adrenal collision tumor was first reported in 1919, approximately 134 cases of adrenal collision tumors (ACTs) have been reported, but their actual prevalence is unknown [7–9]. The components are typically a cortical adenoma, myelolipoma, pheochromocytoma, or metastatic malignant tumor, usually carcinoma [8, 10]. Because of their rarity, the proportion of the components has not been well analyzed [9].

The pathogenesis of ACTs is unknown, but several possible theories have been suggested [9, 11, 12]. The first theory proposes the chance coincidence of two tumors in a contiguous area. The second theory is that a single carcinogenic stimulus alters a particular site and allows the simultaneous occurrence of multiple tumors in proximity. The third one is that the presence of a preexisting tumor may change the local environment, creating ideal conditions for the development of other tumors. Lastly, the small size of the adrenal gland also permits the nearness of multiple tumors.

ACOs are usually clinically silent [4, 13]. In imaging studies, increased and heterogeneous attenuation on CT and the absence of signal intensity loss on opposed-phase MRI are characteristics that can exclude adenomas [14]. Under the microscope, oncocytomas have a diffuse architecture. The tumor cells have abundant granular eosinophilic cytoplasm, confirmed to be rich in mitochondria with IHC study or electron microscopy [15]. In particular, lipofuscin pigments were characteristic in this case. Up to now, atypical features of oncocytomas include brownish pigmentation as well as feeder vessels, keratinization, and pedunculated and lobulated growth patterns [16, 17], but they have not been reported in the adrenal gland.

The prevalence of adrenal ganglioneuromas has been reported to range from 5 to 9.4% in a series of adrenalectomies [18]. Owing to the rarity, most of the available information on this type of tumor has been based on the series of case reports in the literature [6]. Ganglioneuromas usually appear as a well-demarcated homogenous round mass, often surrounding major blood vessels [19]. They seldom recur or metastasize to the regional lymph nodes or distant organs [20].

ACTs, including oncocytomas and ganglioneuromas, have rarely been reported. Only one case showed an oncocytoma with an aldosterone-producing adenoma [21]. Ganglioneuromas have been presented as mixed or composite forms, accompanying myelolipoma or pheochromocytoma [22, 23]. Although both tumors are usually benign, 17.7% of ACOs and 3.7% of adrenal ganglioneuromas show malignant transformation [4, 6]. Therefore, a careful workup with laboratory, endocrine, and imaging studies is essential. If patients present with benign imaging findings and have nonspecific hormonal references, laboratory, endocrine, and imaging studies should be repeated at 6, 12, and 24 months and followed up annually for additional 4 years [24]. Surgery should be considered when the tumor diameter is 4 cm or more, the tumor dimension increases by 1 cm or more, or newly developed hormonal symptoms or abnormal endocrine results appear [6, 24].

In conclusion, both oncocytoma and ganglioneuroma are unusual components of ACTs, and, to the best of our knowledge, the coexistence of these two tumors is unprecedented. Furthermore, a pigmented oncocytoma has not yet been reported in the adrenal gland, and it is also a very rare condition in other sites [16, 17]. Therefore, we present this exclusive case of the coexistence of an ACO and ganglioneuroma in the left adrenal gland [25].

## Figures and Tables

**Table 1:** Data of endocrine tests.

Test	Value	Normal range
RI-aldosterone (erect) (serum) (ng/dL)	**46.5**	2.7–27.2
Renin (erect) (plasma) (ng/mL/hr)	**15.15**	3.95–1.30
Cortisol (serum) (suppressed) (*μ*g/dL)	1.81	<2
ACTH (Plasma) (pg/mL)	2.0	10–50
Metanephrine, total (urine, 24 hrs) (mg/day)	0.3	<0.8
Epinephrine (urine, 24 hrs) (ug/day)	12.0	0–20
Norepinephrine (urine, 24 hrs) (ug/day)	39.0	15–80
VMA (urine, 24 hrs) (mg/day)	3.4	0–8
Epinephrine (plasma) (pg/mL)	49.3	<111 (supine), <141 (standing)
Norepinephrine (plasma) (pg/mL)	107.1	70–750 (supine), 200–1700 (standing)
Metanephrine (plasma) (nmol/L)	0.18	<0.5
Normetanephrine (plasma) (nmol/L)	0.54	<0.9

^*∗*^Significantly increased values are written in bold.

## References

[1] Lau S. K., Chu P. G., Weiss L. M. (2011). Mixed cortical adenoma and composite pheochromocytoma-ganglioneuroma: an unusual corticomedullary tumor of the adrenal gland. *Annals of Diagnostic Pathology*.

[2] Surrey L. F., Thaker A. A., Zhang P. J., Karakousis G., Feldman M. D. (2012). Ectopic functioning adrenocortical oncocytic adenoma (oncocytoma) with myelolipoma causing virilization. *Case Reports in Pathology*.

[3] Sasano H., Suzuki T., Sano T., Kameya T., Sasano N., Nagura H. (1991). Adrenocortical oncocytoma: a true nonfunctioning adrenocortical tumor. *American Journal of Surgical Pathology*.

[4] Bisceglia M. M. D., Ben-Dor D. M. D., Pasquinelli G. M. D. (2005). Oncocytic adrenocortical tumors. *Pathology Case Reviews*.

[5] Enzinger F. M., Weiss S. W. (1995). *Soft Tissue Tumors*.

[6] Shawa H., Elsayes K. M., Javadi S. (2014). Adrenal ganglioneuroma: features and outcomes of 27 cases at a referral cancer centre. *Clinical Endocrinology*.

[7] Meyer R. (1919). Beitrag zur verstandigung uber die namengebung in der geschwulstlehre. *Zentralblatt fur Allgemeine Pathologie*.

[8] Abdullazade I. S., Tezel G. (2012). A rare case of collision tumor: coexistence of adrenocortical adenoma and pheochromocytoma in the same adrenal gland. *Journal of Medical Cases*.

[9] Schwarte L. H., Macari M., Huvos A. G., Panicek D. M. (1996). Collision tumors of the adrenal gland: demonstration and characterization at MR imaging. *Radiology*.

[10] Untch B. R., Shia J., Downey R. J., Carrasquillo J. A., Panicek D. M., Strong V. E. (2014). Imaging and management of a small cell lung cancer metastasis/adrenal adenoma collision tumor: a case report and review of the literature. *World Journal of Surgical Oncology*.

[11] Siddiqi A. J., Miller F. H., Kasuganti D., Nikolaidis P. (2009). Adrenal hemangioma-adenoma: an exceedingly rare adrenal collision tumor. *Journal of Magnetic Resonance Imaging*.

[12] Katabathina V. S., Flaherty E., Kaza R., Ojili V., Chintapalli K. N., Prasad S. R. (2013). Adrenal collision tumors and their mimics: multimodality imaging findings. *Cancer Imaging*.

[13] Bisceglia M., Ludovico O., Di Mattia A. (2004). Adrenocortical oncocytic tumors: report of 10 cases and review of the literature. *International Journal of Surgical Pathology*.

[14] Tirkes T., Gokaslan T., McCrea J. (2011). Oncocytic neoplasms of the adrenal gland. *American Journal of Roentgenology*.

[15] Chang A., Harawi S. J. (1992). Oncocytes, oncocytosis, and oncocytic tumors. *Pathology Annual*.

[16] Say E. A. T., Shields C. L., Bianciotto C., Eagle R. C., Shields J. A. (2012). Oncocytic lesions (oncocytoma) of the ocular adnexa: report of 15 cases and review of literature. *Ophthalmic Plastic and Reconstructive Surgery*.

[17] Di Nicola M., Miserocchi E., Rizzo N., Grantoza C. L., Bandello F., Modorati G. (2013). Atypical presentation of a pigmented oncocytoma of the caruncle: A case report. *Case Reports in Ophthalmology*.

[18] Qing Y., Bin X., Jian W. (2010). Adrenal ganglioneuromas: a 10-year experience in a Chinese population. *Surgery*.

[19] Zhou Y., Liang Q., Ou W.-T., Li Z.-Y., Liu Q.-L. (2015). Laparoscopic resection of primary adrenal ganglioneuroma: a case report and review of the literature. *Oncology Letters*.

[20] Srinivasan R., Sreedharan K., Koliyadan V., Krishnand G., Bhat S. S. (2007). Retroperitoneal ganglioneuroma with lymphnode metastasis: a case report. *Indian Journal of Pathology and Microbiology*.

[21] Terui K., Sakihara S., Kageyama K. (2010). A case of adrenocortical oncocytoma occurring with aldosteronoma. *The Journal of Clinical Endocrinology & Metabolism*.

[22] Merchant S. H., Herman C. M., Amin M. B., Ro J. Y., Troncoso P. (2002). Myelolipoma associated with adrenal ganglioneuroma. *Archives of Pathology and Laboratory Medicine*.

[23] Gorgel A., Cetinkaya D. D., Salgur F. (2014). Coexistence of gastrointestinal stromal tumors (GISTs) and pheochromocytoma in three cases of neurofibromatosis type 1 (NF1) with a review of the literature. *Internal Medicine*.

[24] Young W. F. (2007). The incidentally discovered adrenal mass. *The New England Journal of Medicine*.

[25] Adas M., Koc B., Adas G., Ozulker F., Aydin T. (2014). Ganglioneuroma presenting as an adrenal incidentaloma: a case report. *Journal of Medical Case Reports*.

